# Paraquat, but Not Maneb, Induces Synucleinopathy and Tauopathy in Striata of Mice through Inhibition of Proteasomal and Autophagic Pathways

**DOI:** 10.1371/journal.pone.0030745

**Published:** 2012-01-23

**Authors:** Jonathan Wills, Joel Credle, Adam W. Oaks, Valeriy Duka, Jae-Hoon Lee, Jessica Jones, Anita Sidhu

**Affiliations:** Department of Biochemistry and Molecular and Cell Biology, Georgetown University Medical Center, Washington D.C., United States of America; National Institute of Health, United States of America

## Abstract

*SNCA* and *MAPT* genes and environmental factors are important risk factors of Parkinson's disease [PD], the second-most common neurodegenerative disease. The agrichemicals maneb and paraquat selectively target dopaminergic neurons, leading to parkinsonism, through ill-defined mechanisms. In the current studies we have analyzed the ability of maneb and paraquat, separately and together, to induce synucleinopathy and tauopathy in wild type mice. Maneb was ineffective in increasing α-synuclein [α-Syn] or p-Tau levels. By contrast, paraquat treatment of mice resulted in robust accumulation of α-Syn and hyperphosphorylation of Tau in striata, through activation of p-GSK-3β, a major Tau kinase. Co-treatment with maneb did not enhance the effects of paraquat. Increased hyperacetylation of α-tubulin was observed in paraquat-treated mice, suggesting cytoskeleton remodeling. Paraquat, but not maneb, inhibited soluble proteasomal activity on a peptide substrate but this was not associated with a decreased expression of 26S proteasome subunits. Both paraquat and maneb treatments increased levels of the autophagy inhibitor, mammalian target of rapamycin, mTOR, suggesting impaired axonal autophagy, despite increases in certain autophagic proteins, such as beclin 1 and Agt12. Autophagic flux was also impaired, as ratios of LC3 II to LC3 I were reduced in treated animals. Increased mTOR was also observed in postmortem human PD striata, where there was a reduction in the LC3 II to LC3 I ratio. Heat shock proteins were either increased or unchanged upon paraquat-treatment suggesting that chaperone-mediated autophagy is not hampered by the agrichemicals. These studies provide novel insight into the mechanisms of action of these agrichemicals, which indicate that paraquat is much more toxic than maneb, via its inhibitory effects on proteasomes and autophagy, which lead to accumulation of α-Syn and p-Tau.

## Introduction

Parkinson's disease [PD] is the second-most common neurodegenerative disease, after Alzheimer's disease. Epidemiological studies have linked agrichemicals to an increased risk of PD through rural living, farming, drinking well water, and exposure to agrichemicals used in these settings [1]–[3]. Several agrichemicals can selectively damage dopaminergic neurons, leading to the suggestion of an environmental basis for the development of sporadic PD [4], [5]. Indeed, experimental studies have shown unique sensitivity of dopaminergic neurons to the herbicide paraquat, with other populations of neurons unaffected [3], [6], along with reduced motor activity and dose-dependent losses of striatal dopaminergic nerve fibers [7]. Additional evidence to support paraquat's status as a parkinsonism-inducing toxin comes from data demonstrating up regulation and aggregation of α-synuclein [α-Syn] within substantia nigra neurons in paraquat-treated mice [8]. Maneb is a fungicide and permanent parkinsonism has been reported following chronic occupational exposure to maneb [9]. Some reports have suggested that the toxicity of these agrichemicals is enhanced when used together, and animals treated with paraquat and maneb together showed synergistic reduction in motor activity and greater damage to both striatal nerve terminals and nigral cell bodies, relative to treatment with either agent alone [3], [10]–[12]. Moreover, epidemiological studies have also found increased risk for the development of PD upon exposure to both paraquat and maneb [13].

At the molecular level, the mechanisms of action of paraquat and maneb are not well understood. Paraquat inhibits complex I of the mitochondria resulting in enhanced production of reactive oxidative species, which in turn damages dopaminergic neurons [14], [15]. Additionally, paraquat reduces proteasomal function in DJ-1 deficient mice, impairing clearance of dysfunctional proteins [14]. Maneb causes oxidative stress through inhibition of complex III and inhibits proteasomal activity in cultured cells [16], although its effects on proteasomes *in vivo* are not known.

Several genome-wide studies have identified *SNCA* and *MAPT*, the genes encoding α-Syn and Tau, respectively, as major risk factors in the development of PD [17]–[20] and gene multiplications of α-Syn are causal in the development of PD [21]. We have demonstrated tauopathy in postmortem striata of PD patients, as well as in *in vitro* and *in vivo* toxin and transgenic mouse models of PD [22]–[27]. We found that Tau is abnormally hyperphosphorylated in the striata at the following pathological epitopes: Ser202, Ser262 and Ser396/404. These elevated levels of hyperphosphorylated Tau [p-Tau] were accompanied by increases in aggregated α-Syn [27] and remodeling of the tubulin cytoskeleton [26]. Central to the hyperphosphorylation of Tau was the activation of GSK-3β (glycogen synthase kinase 3 β), through its hyperphosphorylation at Tyr216 [24]–[27]. Both the formation of p-Tau and activation of GSK-3β (p-GSK-3β), was strictly dependent on the presence of α-Syn [22]–[24].

We undertook the current studies to analyze the chronic effects of the agrichemicals, maneb and paraquat, on tauopathy and autophagy in mice. Our results show that paraquat exposure results in increased p-Tau levels in striata, enhanced α-Syn accumulation, elevated p-GSK-3β levels and increased hyperacetylation of α-tubulin. By contrast, maneb did not induce such changes nor did it synergize the toxicity caused by paraquat alone; however, maneb reduced paraquat-mediated increases in p-Tau. Paraquat, but not maneb, caused inhibition of the proteasomal pathway, while both maneb and paraquat caused alterations in key proteins of the autophagy-lysosomal pathway [A-LP], with maneb enhancing some effects of paraquat. Neither agrichemical caused alterations in markers of chaperone mediated autophagy [CMA]. These studies provide for the first time unique insight into the mechanistic processes that occur *in vivo* in the striata upon exposure to agrichemicals.

## Results

### Toxicity of maneb and paraquat in mice

Neither maneb nor paraquat alone caused mortality of mice in this study. However, when combined together, maneb+paraquat showed high levels of toxicity with a mortality rate of 40%. Such high levels of mortality in the combined treatments were also found earlier, where 42% of animal death was reported [28].

### Effect of maneb and paraquat on synucleins, p-GSK-3β and GSK-3β in striata

**Figure 1:**
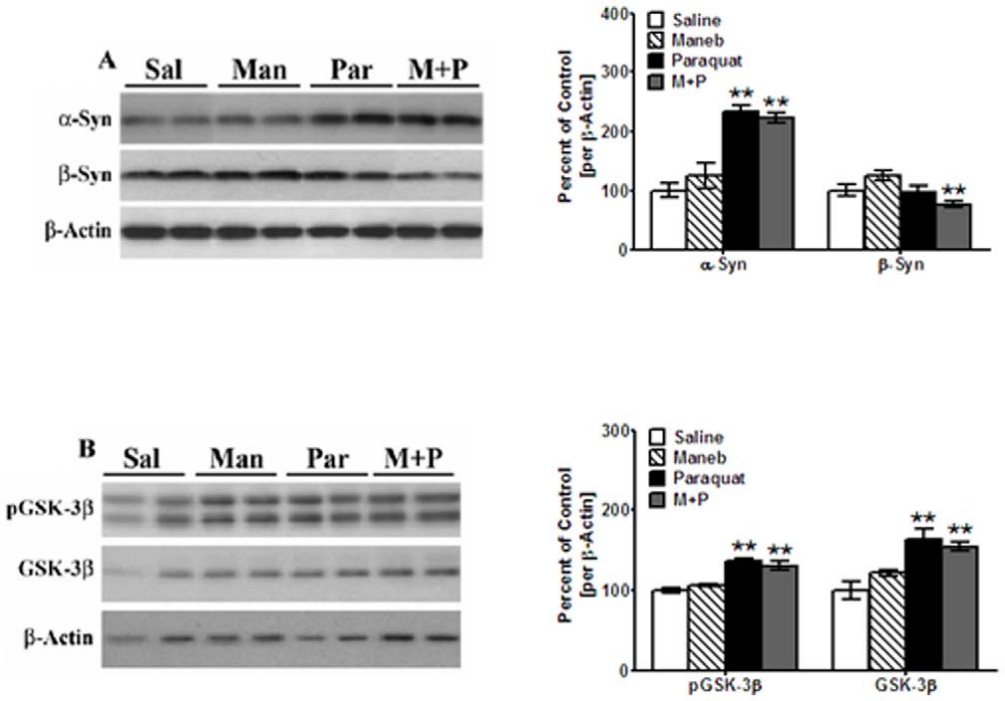
Effect of maneb and paraquat on synucleins, p-GSK-3β and GSK-3β in striata. Striata from saline, maneb, paraquat, and maneb+paraquat injected mice were dissected and homogenized in a modified RIPA buffer containing 1.0% sodium cholate. Cholate-soluble fractions were isolated by centrifugation and analyzed by Western blots, as described under “Materials and Methods”. After exposure to antibodies, blots were stripped and re-probed for other proteins. The blots show representative gels while the bar graphs are composites of blots summarized from all animals (*n* = 5–6). (**A**) α-Syn and β-Syn were expressed relative to β-actin used as a loading control. (**B**) p-GSK-3β levels were probed using antibodies which recognizes a phosphorylation site at Tyr216; both p-GSK-3β and total GSK-3β were expressed relative to β-actin used as a loading control. Values are mean ± SEM as compared to saline-injected animals. Asterisks (**) indicate values significantly different from saline controls (*p*<0.01). Student's t-test was performed to compare saline-treated controls and treatment groups for all data.

Previous studies have shown that maneb and paraquat induced parkinsonian behavior in mice and that maneb synergistically enhanced the effects of paraquat when both these agents were used together [10]–[12], but the biochemical mechanisms underlying such induction has not been previously reported. To study the effects of these agrichemicals, mice were chronically injected twice a week for 6 weeks with maneb or paraquat alone, or with maneb+paraquat, as described under Methods. Striata were isolated, lysed in sodium cholate, and both cholate-soluble and cholate-insoluble fractions were used for analyses. Since there were no significant changes in any of the proteins in the cholate-insoluble fractions, only the data from the cholate-soluble fractions are presented below in Figure 1.

Treatment of mice with maneb failed to cause any significant increases [24%, *p*>0.05] in the expression levels of α-Syn [M_r_ 17 kDa] in cholate-soluble fractions, compared to control saline-treated mice [Fig. 1A]. By contrast, paraquat caused a significant [*p*<0.01] increase [of 133%] in the levels of α-Syn in the striata, compared to control saline-treated mice. In mice injected with maneb+paraquat, significant [*p*<0.01] increases [121%] in α-Syn levels were also observed compared to control mice. Since this increase was not significantly different than that obtained using paraquat alone, this suggests that maneb did not enhance the toxicity of paraquat in the induction of synucleinopathy.

We also examined the levels of β-synuclein [β-Syn, M_r_ 16∼kDa] in cholate-soluble fractions [Fig. 1A]; β-Syn antagonizes the effects of α-Syn and exerts a protective effect on neurons, by counteracting α-Syn toxicity [29]. Neither maneb nor paraquat alone showed significant changes in β-Syn levels (22% increase and 2% decrease compared to control saline-treated mice, respectively, *p*>0.05). When combined, however, maneb+paraquat significantly decreased β-Syn expression levels (by 28%, *p*<0.05).

We next measured changes in p-GSK-3β, a major kinase known to phosphorylate Tau, whose autophosphorylation at Tyr216 causes activation of the kinase [Fig. 1B]. The antibody against p-GSK-3β also recognizes p-GSK-3α, detectable as a band of M_r_∼52 kDa visible above p-GSK-3β; in our studies, only the lower band, corresponding to p-GSK-3β [M_r_ of 46 kDa], was used to calculate for levels of this protein. In maneb-treated mice, increases [7% compared to control saline-treated mice, *p*>0.05] in p-GSK-3β levels were not observed in striata. In paraquat-treated mice, however, we saw elevated levels [of 36%] in p-GSK-3β that were highly significant [*p*<0.01, Fig. 1B]. Similarly, in mice treated with maneb+paraquat, we also saw higher levels of p-GSK-3β [30%] which were significantly [*p*<0.01] different from control mice; maneb treatments did not synergize the activation of p-GSK-3β seen by paraquat alone.

High levels of total non-phosphorylated GSK-3β were also seen in paraquat and maneb+paraquat treated animals [Fig. 1B]. Thus, in maneb-treated mice, total GSK-3β levels were unchanged [22% increase compared to control, saline-treated mice, *p*>0.05]. In paraquat-treated mice, total GSK-3β levels were increased by 64% [*p*<0.01], whereas in maneb+paraquat animals the increase was 55% [*p*<0.01]. These increases in total GSK-3β are likely to be a result of the chronic treatments over a 6 week period, such that increases in total GSK-3β offset the observed increases in p-GSK-3β. That maneb failed to enhance p-GSK-3β or total GSK-3β levels when used alone, or when used in combination with paraquat, further indicates lack of toxicity of maneb in mice striata and lack of synergy between the two compounds, respectively.

### Effect of maneb and paraquat on expression levels of hyperphosphorylated Tau in striata

**Figure 2:**
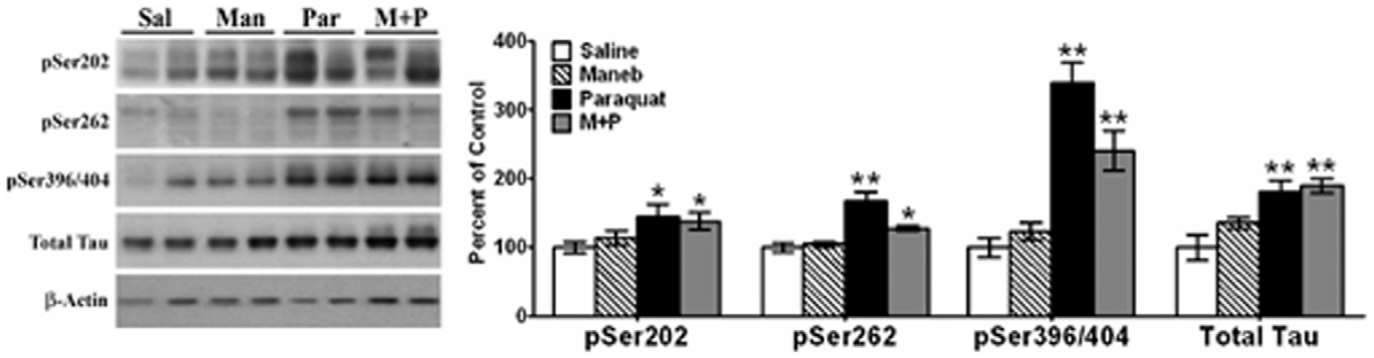
Effect of maneb and paraquat on expression levels of hyperphosphorylated Tau in striata. The blots show representative gels while the bar graphs are composites of blots summarized from all animals (*n* = 5–6). p-Tau and Total Tau were probed using antibodies specific for pSer202, pSer262, pSer396/404, and Tau-5, respectively, and expressed relative to β-actin used as loading control. Values are mean ± SEM as compared to saline injected control. Asterisks (*, **) indicate values significantly different from saline controls (*p*<0.05, *p*<0.01, respectively). Student's t-test was performed to compare control and treatment groups for all data.

Tau is a microtubule-stabilizing protein located within axons in neurons, whose hyperphosphorylation at certain sites is known to be pathological, leading to dissociation of Tau from microtubules, microtubule destabilization, loss of axonal transport and neuronal degeneration [30]–[33]. We examined p-Tau hyperphosphorylated at Ser202 [observed as a doublet with M_r_ of 50 and 56 kDa], and found no significant changes [14% increase, *p*>0.05] in striata of maneb-treated mice [Fig. 2]. By contrast, in paraquat-treated mice, a significant [*p*<0.01] increase [of 67%] in pSer202 Tau was observed, which remained significantly [*p*<0.05] elevated in maneb+paraquat-treated mice [increase of 38%]. pSer262 levels, detected as a smeary series of bands with M_r_ of 50–60 kDa, were unchanged in maneb-treated mice when compared to saline-treated control mice [Fig. 2], while in paraquat-treated mice a significant increase [67% increase, *p*<0.01] was seen. In maneb+paraquat-treated mice, levels of pSer262 remained elevated [28%, *p*<0.05]. pSer396/404 Tau levels [Fig. 2], probed by the PHF-1 antibody and detected as a single band with M_r_ of 50 kDa, were increased in maneb-treated mice [23%] but the increase was not significant [*p*>0.05]. In paraquat-treated mice very high levels of pSer396/404 were seen [240% increase, *p*<0.01], and remained high in maneb+paraquat mice [141%, *p*<0.01].

We also examined total Tau levels, detected as a single band with M_r_ of ∼55 kDa [Fig. 2], and found Tau levels to be increased in maneb-treated mice [23%], but the increase was not significant [*p*>0.05]. In paraquat-treated mice, total Tau was increased [81%], while in maneb+paraquat-treated mice, Tau increased by 90%, and both increases were highly significant [*p*<0.01]. It should be noted that when either cells [22]–[24] or mice [unpublished observations] are acutely treated with toxins, this does not lead to changes in total Tau levels, due to lack of long-term effects on microtubules. However, in the current studies, when mice were chronically treated with toxins which affects microtubule stability [see below], this leads to an overall increase in total Tau, likely due a feed-back mechanism causing an increase in synthesis of total Tau to compensate for stabilization of microtubules. The pattern of increases in p-Tau and total Tau levels in these mice indicates that whereas paraquat treatments significantly increases tauopathy in striata of mice, maneb treatments do not, nor does maneb augment the tauopathy caused by paraquat alone. These findings further support our contention that when microtubules are destabilized under chronic conditions of treatments, total Tau increases in a compensatory manner, similar to the increase in total GSK-3β seen above in Figure 1B, to offset a long-term sustained increase in p-GSK-3β, which eventually leads to microtubule stability.

### Effect of maneb and paraquat on acetylation of α-tubulin

**Figure 3:**
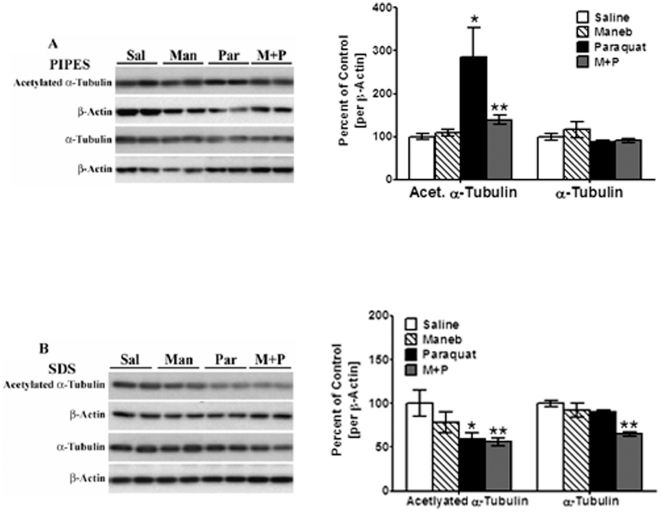
Effect of maneb and paraquat on acetylation of tubulin. Using the PIPES method to extract cytoskeleton-free fractions (**A**, PIPES) by centrifugation, pellet fractions were solubilized in SDS to re-suspend cytoskeleton-associated fraction (**B**, SDS). Both cytoskeleton-free and cytoskeleton-associated fractions were analyzed by Western blots. The blots show representative gels while the bar graphs are composites summarized from all animals (*n* = 5–6). Acetylated-α-tubulin was probed using antibodies specific for acetylated α-tubulin and expressed relative to β-actin used as loading control. Values are mean ± SEM as compared to saline injected control. Asterisks (*, **) indicate values significantly different from saline controls (*p*<0.05, *p*<0.01, respectively). Student's t-test was performed to compare control and treatment groups for all data.

Earlier studies have shown that an excess of Tau protein increases hyperacetylation of α-tubulin, through an inhibitory effect on histone deactylase 6, whose substrates include tubulin [34]. While α-tubulin acetylation enhances microtubule stability, excessive acetylation can impair retrograde axonal transport [35]. We decided to investigate whether the increased levels of Tau and p-Tau seen in striata of paraquat treated mice results in changes in the state of α-tubulin acetylation. Striata from mice were extracted in PIPES buffer and centrifuged to isolate cytoskeleton-free (PIPES) and cytoskeleton-associated (SDS) fractions, as described in Methods, and levels of acetylated α-tubulin were examined in both fractions. Under normal conditions, α-tubulin levels are low in cytoskeleton-free fractions; however, when microtubules are destabilized, elevated levels of free α-tubulin appear in cytoskeleton-free fractions. While there were no changes in total α-tubulin present in the cytoskeleton-free fraction, large increases in acetylated free α-tubulin [of 185%, *p*<0.05] were seen in paraquat-treated mice, with smaller increases [∼40%, *p*<0.05] in mice treated with both maneb and paraquat [Fig. 3A]. Such increases in free α-tubulin represent destabilization of α-tubulin. Maneb alone failed to induce any changes in acetylated α-tubulin in the cytoskeleton-free fractions, suggesting that this toxin did not cause destabilization of microtubules. The increases in acetylated α-tubulin in cytoskeleton-free fractions were accompanied by a corresponding decrease in acetylated α-tubulin in cytoskeleton-associated fractions of paraquat-treated and maneb+paraquat-treated mice [40 and 44%, respectively, *p*<0.01, Fig. 3B]. Interestingly, total α-tubulin levels were also decreased in maneb+paraquat-treated mice [35%, *p*<0.01] [Fig. 3B].

### Effect of maneb and paraquat on proteasomal activity

**Figure 4:**
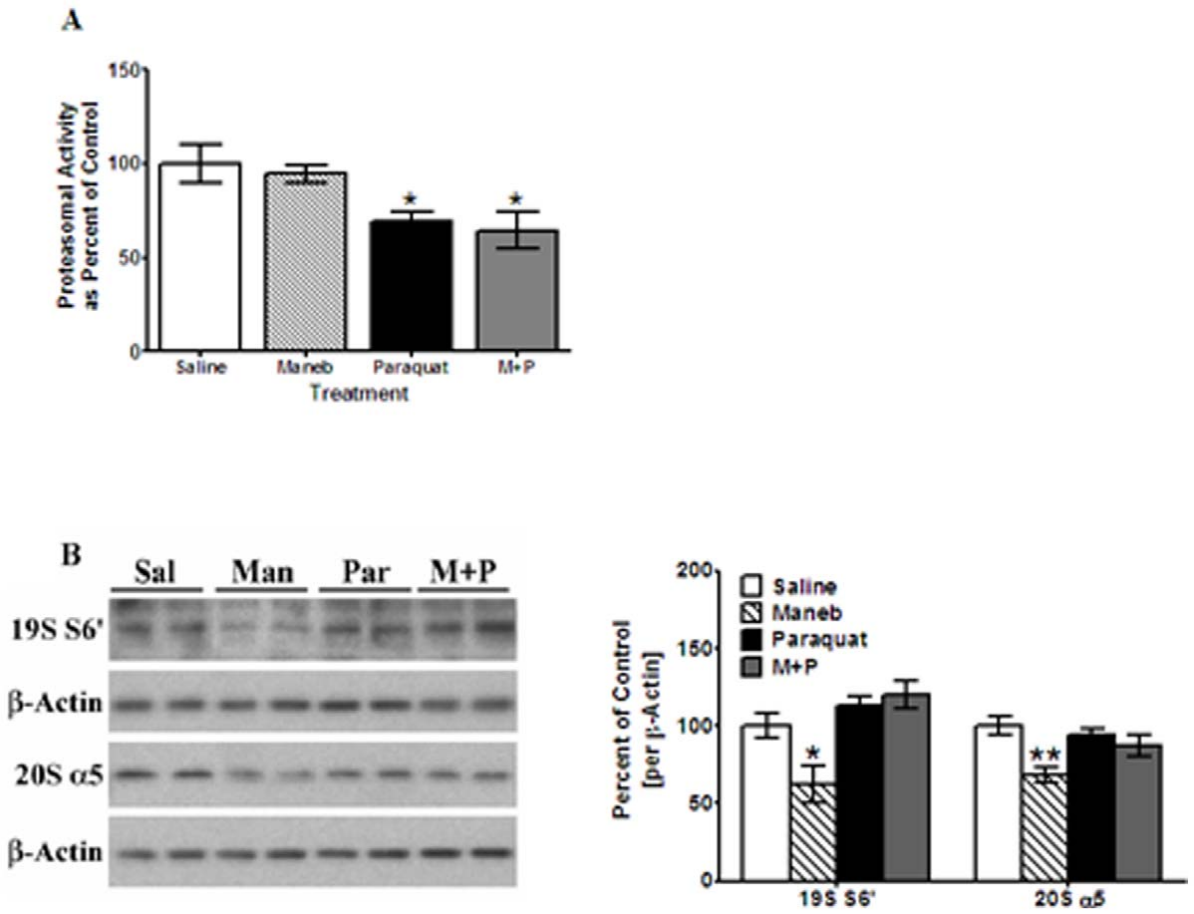
Effect of maneb and paraquat on proteasomal activity. (**A**) Prior to setting up the assay protocol, brain samples were extracted by sodium cholate, and sample assays from both insoluble and soluble fractions ranged from 579–3845 units and 31,000–78,000 units, respectively, indicating that the solubilization protocol was working optimally. Aliquots from soluble extracts were used to measure 26S proteasomal activity as described under “Materials and Methods”. Lactacystin-sensitive digestion of the model substrate suc-LLVYAMC was used to measure 26S proteasome activity, expressed via AMC fluorescence. (**B**) Samples were further homogenized in a modified RIPA buffer containing 1.0% sodium cholate, and analyzed by Western blots. After exposure to initial antibodies, blots were stripped and probed for other proteins. The blots show representative gels while the bar graphs are composites summarized from all animals (*n* = 5–6). Proteasomal proteins 19S and 20S were probed using antibodies specific for the 19S 6S′ subunit and 20S α5 subunit, and expressed relative to β-actin used as loading control. Values are mean ± SEM as compared to saline injected control. Asterisks (*, **) indicate values significantly different from saline controls (*p*<0.05, *p*<0.01, respectively). Student's t-test was performed to compare control and treatment groups for all data.

The primary pathway by which proteins are cleared from cells is through proteasomes, and the increased accumulation of α-Syn and p-Tau suggested to us that proteasomal activity may be impaired by the agrichemicals. Studies have shown that paraquat reduces proteasomal activity by 30% in wild type and DJ-1 deficient mice [14]. Maneb was also found to inhibit proteasomal activity [16], but these findings were obtained *in vitro* in cultured cells and its effect on proteasomes *in vivo* is not known. For these reasons, we decided to examine proteasomal activity in striata of these mice [Fig. 4A]. Lactacystin-sensitive digestion of the model substrate suc-LLVYAMC was used to measure activity of the 20S proteolytic component of the 26S proteasome. Cleavage of LLVY is independent of ubiquitylation or 19S chaperone activity and so is considered a direct measure of 26S proteolytic activity. Preliminary experiments demonstrated that greater than 80% of the total 26S proteolytic activity was extracted into soluble fractions by our extraction procedure.

In maneb-treated mice, 26S proteolytic activity was not significantly different [95% of control, *p*>0.05%] than that obtained in saline-treated mice [Fig. 4B]. In paraquat-treated mice, 26S proteolytic activity was significantly reduced [by 40%, *p*<0.05] compared to control saline-treated mice. The 26S proteolytic activity in maneb+paraquat-treated mice was also significantly [*p*<0.05] reduced [by 35%] compared to control mice and was comparable with the activity seen in mice treated with paraquat alone.

In order to ascertain whether the reduction in 26S proteolytic activity was due to alterations in the subunits of the proteasome, we examined the protein levels of representative subunits of both the 19S and 20S components (S6′ and alpha 5, respectively) of the 26S proteasome by Western blots [Fig. 4B]. While we consistently observed alterations in total proteasome levels in certain groups, these did not correlate with reductions in 26S proteolytic activity and measured by LLVY cleavage. Both the 19S and 20S subunits were detected at similarly reduced levels in maneb treated mice (38% and 32%, respectively) relative to control samples. The similar reduction in both the 19S and 20S subunits suggests that there were no substantial alterations in 26S assembly. While this reduction was statistically significant [p<0.05], it was not associated with a decease in proteolytic activity. In paraquat-treated mice, 19S S6′ levels were increased by 13%, while in maneb+paraquat-treated animals, an increase of 20% was observed. Levels of the 20S alpha 5 subunit in paraquat and maneb+paraquat-treated mice were decreased by 5 and 13%, respectively. However, none of the changes in proteasomal expression levels associated with paraquat or paraquat+maneb treated mice were statistically significant [*p*>0.05]. These data indicate that decreases in proteasomal proteolytic activity observed upon treatment with paraquat were not due to loss of either the 19S or 20S components or changes in the assembly of the 26S proteasome. Instead, paraquat appears to have a direct inhibitory effect on the proteolytic activity of the 20S component of the proteasome.

Additionally, since both α-Syn and p-Tau bind to proteasomes reducing its activity [25], [40], the decreases in proteasomal activity upon paraquat or paraquat+maneb treatment, may at least be due in part to the direct inhibitory effects of α-Syn and p-Tau, which are produced by paraquat. Moreover, since maneb treatments do not induce increases in α-Syn and p-Tau, the lack of changes in proteasomal activity in maneb-treated fractions may be explained by the absence of α-Syn and p-Tau.

### Effect of maneb and paraquat on axonal autophagy and chaperone-mediated autophagy

When proteasomes are inhibited, misfolded proteins such as α-Syn and p-Tau start to aggregate and accumulate at nerve terminals. This in turn triggers the activation of both chaperone mediated autophagy [CMA] and the autophagic lysosomal pathway [A-LP]. Proteins are then conjugated to individual heat shock proteins or packaged into autophagosomes, where they are transported back to cell soma containing lysosomes and Lamp2a, through retrograde transport by dynein along MTs [36]–[39], [41]. Since the effect of these agrichemicals on axonal autophagy is not known, we decided to examine the major components of axonal autophagy in striatal lysates.

**Figure 5:**
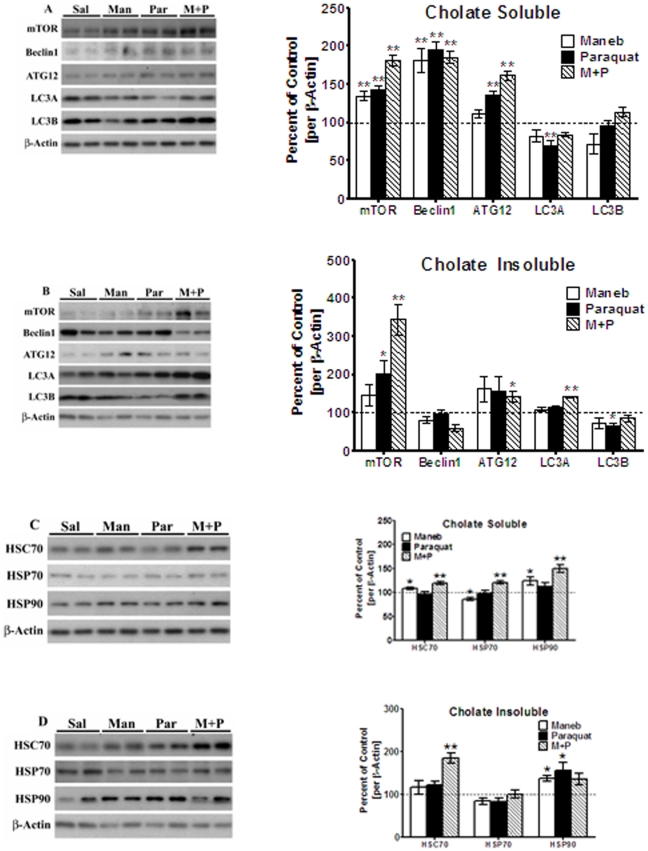
Effect of maneb and paraquat on autophagic pathway. Striata from saline, maneb, paraquat, and maneb+paraquat injected mice were homogenized in a modified RIPA buffer containing 1.0% sodium cholate, and separated into cholate-soluble and cholate-insoluble fractions and analyzed by Western blots, as described under “Materials and Methods”. The blots show representative gels while the bar graphs are composites summarized from all animals (*n* = 5–6). (**A**) Autophagy pathway proteins in the soluble and (**B**) insoluble supernatant were probed using antibodies specific for mTOR, Beclin1, and Atg12, and expressed relative to β-actin used as loading control. (**C**) LC3 I (upper band) and LC3 II (lower band in longer exposure blot image) were probed in cholate soluble fractions using LCB antibodies, and the ratio of LC3 II to LC3 I was assessed to measure autophagic flux. (**D**) Chaperone proteins in the cholate soluble and (**E**) cholate insoluble fractions were probed using antibodies specific for Hsc70, Hsp70, and Hsp90, and expressed relative to β-actin used as loading control. Values are mean ± SEM as compared to saline injected control. Asterisks (*, **, ***) indicate values significantly different from saline controls (*p*<0.05, *p*<0.01, *p*<0.001,respectively). Student's t-test was performed to compare control and treatment groups for all data.

In cholate-soluble fractions, both maneb and paraquat significantly [p<0.01] increased [35 and 43%, respectively] levels of the mammalian target of rapamycin [mTOR], an overall inhibitor of A-LP; when used together, maneb+paraquat augmented the increase in mTOR levels [81%, p<0.01] [Fig. 5A]. Elevated levels of mTOR were also detected in cholate insoluble fractions treated with paraquat alone [Fig. 5B, 103%, p<0.05] or maneb+paraquat [Fig. 5B, 245%, p<0.01]. Significant [p<0.01] increases in levels of beclin 1, an inducer of autophagy, were also seen in cholate-soluble fractions under all three treatment paradigms, with increases ranging from 81–95% [Fig. 5A]. In cholate-insoluble fractions, however, no significant changes in beclin 1 levels were observed, and in fact small decreases were seen [Fig. 5B]. Levels of Atg12, another inducer of A-LP, were also increased in cholate-soluble extracts of paraquat and paraquat+maneb-treated striata [36 and 62%, respectively, p<0.01], but not in maneb-treated animals [Fig. 5A]. In cholate insoluble fractions [Fig. 5B], significant increases in Atg12 levels were noted only for paraquat+maneb treatments [45%, p<0.05]. In the A-LP pathway, a single inhibitor of this pathway exists, mTOR, while numerous activators of the pathway, beclin 1, Atg5, Atg7 and many other Atg proteins are also found. Our data suggests that despite increases in beclin 1 and Atg12, the sharp increases in mTOR may override the stimulatory effects of these other proteins, leading to an overall mTOR-mediated inhibition of A-LP in this system.

To gain further understanding of whether autophagosomes are formed, which would enable us to determine whether A-LP is inhibited, we examined levels of the microtubule-associated protein 1 light chain 3 [LC3], which is an essential component of the autophagosome [Fig. 5C]. In particular, newly synthesized LC3 is proteolytically cleaved to a smaller protein [LC3 I, M_r_∼16 kDa] which becomes lipidated [LC3 II, M_r_∼14 kDa] and is then inserted into the autophagosome.

Ratios of lipidated LC3–II to cytosolic LC3-I provide an important indicator for monitoring the autophagic activity (or flux) [38]. We therefore measured the LC3 II to LC3 I ratio in cholate soluble fractions using the LC3B antibodies [Fig. 5C]. It should be noted that LC3 II bands are not very stable and when present, are often present as small punctate proteins or faint bands. From Figure 5C, only low and faint levels of LC3 II bands were visible, below the stronger, darker LC3 I bands. When ratios were calculated, the ratio was significantly depressed in all treatment groups [maneb, 42%, p<0.001; paraquat, 24%, p<0.05; maneb+paraquat, 23%, p<0.05], indicative of a lowered level of autophagic flux compared to the saline-treated group (Fig. 5C).

We next examined components of chaperone-mediated autophagy. Levels of the heat-shock cognate protein 70 [Hsc70] were only modestly increased in cholate-soluble fractions [Fig. 5D] from maneb and maneb+paraquat treated mice [8–19%, *p*<0.05], while in the cholate-insoluble fractions [Fig. 5E], this protein was greatly increased [by 85%, p<0.01] in the paraquat+maneb treated mice. Heat-shock protein 70 [Hsp70] levels were reduced in cholate-soluble extracts from maneb treated mice, while a small but significant [p<0.01] increase of 21% was seen in paraquat+maneb treated animals [Fig. 5D]. No significant changes were seen in Hsp70 levels in cholate-insoluble fractions [Fig. 5E]. When Hsp90 levels were examined [Fig. 5D], significant [*p*<0.01] increases were seen in soluble fractions from maneb [35%], paraquat [43%] and maneb+paraquat [81%] treated mice. Increased levels of Hsp90 were also seen in insoluble fractions for maneb and maneb+paraquat treatments [39% and 55%, respectively, *p*<0.05, Fig. 5E]. Together, these data suggest that levels of the heat shock proteins are increased after maneb and paraquat treatments.

### Immunohistochemical co-localization in Striatum and Midbrain

**Figure 6:**
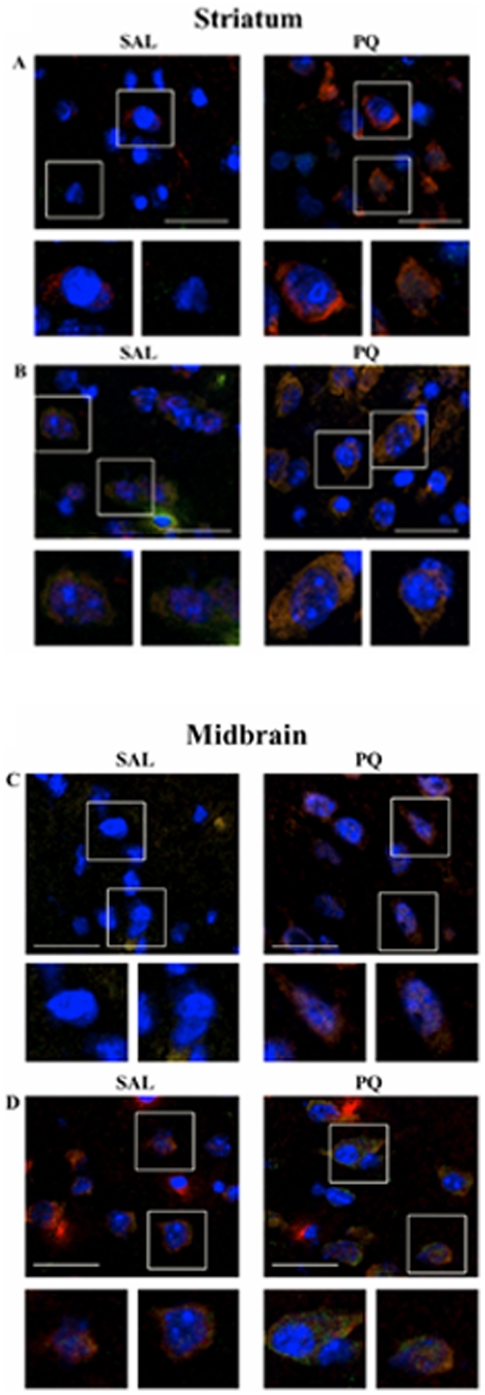
Immunohistochemical co-localization in striatum and midbrain. Representative striatum (**A–B**) and midbrain (**C–D**) sections double immunostained for α-Syn (Red), with PHF-1 Tau (Green), or mTOR (Green) and nuclei with DAPI (Blue). Single antibody images from saline (Sal, top panel) and paraquat (PQ, bottom panel) treated mice shown at 40×. White boxes indicate highlighted areas shown at greater magnification in side panels, far right. (**A**) Striatum immunostained with α-Syn (Red), PHF-1 Tau (Green), and DAPI (Blue). (**B**) Striatum immunostained with α-Syn (Red), mTOR (Green), and DAPI (Blue). (**C**) Midbrain immunostained with α-Syn (Red), PHF-1 Tau (Green), and DAPI (Blue). (**D**) Midbrain immunostained with α-Syn (Red), mTOR (Green), and DAPI (Blue). Scale bar 10 µm.

To further explore the relationship between α-Syn, p-Tau and autophagy, dual immunohistochemical [IHC] staining was conducted, and co-localization of α-Syn with mTOR or PHF-1 Tau (p-Tau) was evaluated in striatal and midbrain sections of paraquat-treated or control mice [Fig. 6]. In both striata [Fig. 6A] and midbrain sections [Fig. 6C] increased immunostaining, with co-localization, was seen for α-Syn and p-Tau in paraquat-treated mice compared to saline-treated mice. Dual staining of α-Syn and mTOR in striatal [Fig. 6B] and midbrain sections [Fig. 6D] showed elevated levels of both proteins that were co-localized with one another in the paraquat-treated versus saline-treated mice. These results are consistent with the Western blot analysis of Fig. 5A, showing increased levels of mTOR. mTOR showed a more punctate staining pattern in the striatal samples, whereas in the midbrain, the staining was more diffuse. It is interesting to note that some mTOR staining was observed in the saline-treated mice for both striatal and midbrain regions, with a peri-nuclear localization. This is in contrast to the paraquat-treated sections in which α-Syn and mTOR is seen being distributed more in the cytoplasmic cell body.

### Impaired axonal autophagy in postmortem striata of Parkinson's disease

**Figure 7:**
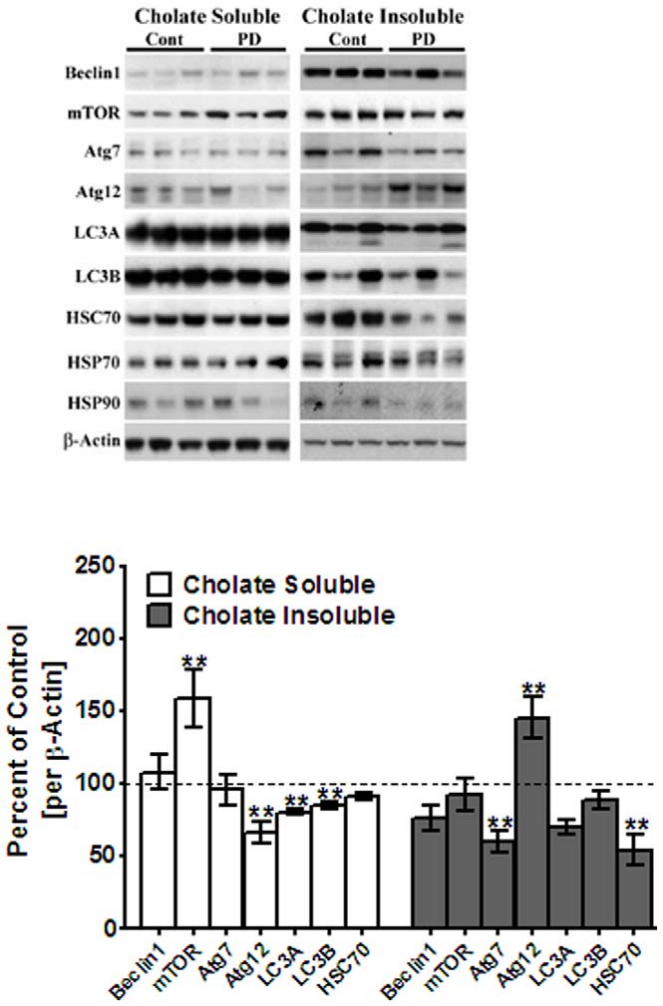
Western blot analyses of striata from PD brains and age-matched controls. (A) Cholate soluble and cholate insoluble fraction from PD striata and age-matched controls were analyzed by Western blots. Blots show representative gels while bar graphs are composites summarized from all postmortem tissues (*n* = 10–17). All proteins were expressed relative to β-actin used as loading controls. (B) LC3 II/LC3 I ratios were calculated to assess autophagic flux. Values are mean ± SEM as compared to age-matched control tissue. Asterisks (*, **) indicate values significantly different from controls (*p*<0.05, *p*<0.01, respectively). Student's t-test was performed to compare control and PD samples for all data.

No studies have been conducted on axonal autophagy in nerve terminals in PD. To assess if axonal autophagy is impaired in PD, we examined mTOR and major proteins involved in autophagosome formation in postmortem striata from PD patients and age-matched non-diseased controls [N = 10–17 each]. Striata were solubilized in 1% sodium cholate and cholate-soluble and cholate-insoluble fractions were isolated and examined by Western blots [Fig. 7A]. In cholate-soluble supernatant fractions, there was a significant increase [63%, *p*<0.01] in mTOR levels in PD striata compared to controls, while in cholate-insoluble fractions, mTOR levels were not changed [Fig. 7A]. In cholate-soluble fractions, we also observed decreases of ∼35% [*p*<0.01] in Atg12 levels. There were no significant changes in levels of beclin 1 or Atg7 in PD brains compared to controls. There were also no changes in levels of markers of chaperone-mediated autophagy, Hsc70, Hsp70 and Hsp90.

In striatal cholate-insoluble fractions [Fig. 7A], Atg7 levels were significantly reduced [by 40%, *p*<0.01], whereas Atg12 levels were increased [50%, *p*<0.01] in PD. Interestingly, Hsc70 levels were decreased [50%, *p*<0.01] in pellet fractions, while Hsp70 and Hsp90 levels were unchanged.

We also calculated the LC3 II to LC3 I ratios in human PD striatum [Fig. 7B] and found that this ratio was significantly reduced [40%, *p*<0.001] in PD brain compared to controls, indicating reduced autophagic flux.

Together, the combined data suggest an overall inhibition of axonal autophagy via increased levels of mTOR, along with an impaired ability to form autophagosomes and reduced chaperone-mediated autophagy by Hsc70.

## Discussion

The data presented here shows for the first time that paraquat induces a broad spectrum of pathological changes in striata which includes synucleinopathy, tauopathy, destabilization and hyperacetylation of microtubules, and inhibition of both proteasomal and autophagic pathways. By contrast, the effects of maneb were much milder, causing only a modest inhibition of A-LP and a decrease in autophagic flux. Moreover, apart from increasing mTOR levels, maneb also did not appear to enhance the pathology of paraquat, which is in contrast to previous reports suggesting that maneb synergizes paraquat-mediated toxicity [10], [11]. Yet, maneb and paraquat together was much more toxic to animals than either agrichemical used alone, resulting in ∼40% mortality of animals. Together, these data imply that paraquat is much more toxic than maneb, inducing changes in striata which mimic those seen in sporadic PD [25], which include synucleinopathy, tauopathy, microtubule destabilization, inhibition of proteasomes and A-LP.

The tauopathic changes in striata of mice induced by paraquat, indexed by elevated levels of pSer202, pSer262 and pSer396/404, are the same sites that we have previously shown to be hyperphosphorylated in PD postmortem striata [25]. It is now well-established that hyperphosphorylation of Tau at such pathological epitopes can initiate the neurodegenerative process, since hyperphosphorylation reduces the ability of Tau to bind to microtubules, leading to enhanced destabilization of the microtubular network, disruption of axonal transport, with eventual degeneration of nerve terminals [30]–[32]. In particular, hyperphosphorylation at Ser262 detaches Tau from MTs [33] and hyperphosphorylation at Ser396/404 promotes self assembly of Tau to form aggregates [30]–[32]. In mice striata, paraquat treatment caused increased levels of soluble total Tau to be present in cholate-soluble extracts, along with increased levels of hyperacetylated α-tubulin in cytoskeleton-free fractions and reduced levels of hyperacetylated α-tubulin in cytoskeleton-associated fractions, suggesting remodeling of the microtubule cytoskeleton.

Paraquat also caused increases in α-Syn levels in striata of mice, but the additional presence of maneb did not augment this increase. Moreover, paraquat-treatment led to increased activity of p-GSK-3β, hyperphosphorylated at Tyr216. p-GSK-3β is a major kinase involved in the hyperphosphorylation of Tau at numerous sites, and its activation and role in mediating Tau hyperphosphorylation in PD has now been established through studies we have conducted in postmortem PD striata [25] and in *in vitro* and *in vivo* models of PD [24], [27]. Interestingly, treatment of mice with both MPTP [22] and paraquat [the current study] leads to similar changes in α-Syn, p-GSK-3β and p-Tau, and this may be due to a high degree of overlap in the structural homology and mechanism of action shared by these chemicals. In contrast to paraquat, maneb failed to cause increases in levels of α-Syn or to activate GSK-3β, nor did maneb augment paraquat-induced increases in these proteins. Moreover, the effect of maneb on paraquat with regard to p-Tau formation suggests that maneb may act to reduce paraquat-mediated tauopathy. Thus, maneb significantly reduced levels of both pSer262 and pSer396/404 in mice co-treated with paraquat. Although this did not change the overall levels of soluble total Tau, the additional presence of maneb diminished the levels of cytoskeleton-free acetylated α-tubulin.

Furthermore, unlike paraquat, maneb did not directly inhibit 26S proteolytic activity *in vivo*, nor did it exacerbate the direct inhibitory effect of paraquat on this activity. Maneb treatment resulted in a moderate but significant decrease in 26S steady state expression levels, as measured by representative components from the 19S and 20S subunits, which was alleviated by co-treatment with paraquat. However, this reduction was not accompanied by a reduction in ubiquitin-independent 26S proteolytic activity on a peptide substrate, indicating that the level of proteolytically active proteasomes was not directly related the total proteasome levels in these samples. Control of 26S proteasome expression is complex, and our data suggest that maneb has a direct or indirect effect on this process. Small increases in proteasomal components seen in paraquat treated samples may be the result of compensatory changes in response to proteolytic inhibition and may account for the alleviation of the effect of maneb on expression. We can not rule out the possibility that decreases in steady state levels of 26S proteasome in maneb-treated mice could lead to decreased efficiency of degradation of specific ubiquitylated substrates, which may partially account for the extreme toxicity seen in doubly-treated mice. Toxicity would arise from a direct effect of paraquat on proteolytic activity, something that would affect all substrates, and an effect of maneb on 26S expression level, which may affect some substrates more than others. This will be an important area of future study. Whatever the mechanism, proteasomal inhibition is known to be toxic to cells, as it prevents cells from processing proteins targeted for degradation. Decreases in proteasomal activity have been previously noted in PD substantia nigra [41] and PD striata [25] which may partially account for accumulation of both α-Syn and p-Tau seen in dopaminergic neurons, which then interact with one another leading to formation of Lewy bodies and Lewy neurites.

Additional differences between maneb and paraquat were seen with regard to their effects on autophagy. In neurons, when proteasomes are inhibited, the A-LP is activated as a means of degrading proteins originally targeted to the proteasome [37]. Both maneb and paraquat increased levels of mTOR, an inhibitor of autophagy [36]–[39], suggesting that the A-LP is dysfunctional in these mice. Interestingly, maneb augmented the increase in mTOR levels elicited by paraquat alone, suggesting that maneb+paraquat produces the greatest inhibition of ALP than either of the agrichemicals used alone. The high levels of mTOR may lead to compensatory increases in levels of other components of the A-LP, such as beclin 1, a major initiator of autophagy [36]–[39], as well as Atg12, a marker of the early stages of A-LP and a protein important in autophagosome formation. It is noteworthy that we found reduced autophagic flux upon agrichemical treatment and these findings are at odds with those of others [42], who found increases in LC3 proteins associated with autophagic vacuoles in cultured SH-SY5Y cells treated with paraquat. This may be due to differences that are intrinsic to *in vitro* versus *in vivo* models, or, alternatively, may be due to differences in the effect of acute treatments [42] and chronic treatments [our study] on LC3 function. Our IHC findings in midbrain regions indicate that impaired autophagy, as indexed by increased levels of mTOR in these regions, is not restricted to striata, but is also evident in cell soma.

CMA is an important pathway by which aggregates of proteins are cleared from nerve terminals [36]–[39]. In this pathway, proteins at nerve terminals destined for degradation bind to heat-shock chaperone proteins, such Hsc70, Hsp70 and Hsp90 proteins, and are then delivered to lysosomes in the cell soma through retrograde transport on microtubules [43], [44]. With the exception of maneb, in paraquat and paraquat+maneb treated mice, the levels of the three heat shock proteins measured, Hsc70, Hsp70 and Hsp90, either increased or were unchanged compared to control mice; in maneb-treated mice, Hsp70 levels were modestly reduced, while both Hsc70 and Hsp90 levels were increased. This suggests that the CMA pathway in these mice is not likely to be impaired, and may function normally. Indeed, CMA up regulation and increases in Hsp70 have been previously observed in mice after a single injection of paraquat [45]. However, in our studies, it is not clear whether chaperone proteins with their cargoes reach the cell soma, since such retrograde transport of CMA is dependent on microtubules, and the high levels of acetylated α-tubulin seen after PQ treatments may hamper appropriate delivery of these cargoes to the lysosome in the cell soma. Indeed, hyperacetylation of α-tubulin has been previously shown to lead to impaired microtubular transport of mitochondria [35], and the hyperactylated α-tubulin observed after paraquat treatment may similarly hamper CMA. This, together with impaired proteasomal activity and A-LP, may be the major reasons leading to accumulation of α-Syn and p-Tau in paraquat-treated striata.

Our findings in paraquat-treated mice parallel those seen in PD postmortem striata, where we observed high levels of mTOR, suggesting impairment of ALP in nerve terminals. Moreover, we also observed decrease in the LC3 II to LC3 ratio in cholate-soluble extracts of PD striata. Unlike our findings in paraquat-treated mice, PD striata demonstrated reductions in Atg7 and Atg12 proteins, further indicating that ALP may be impaired in PD compared to age-matched non-diseased controls. Although neither Hsp70 nor Hsp90 levels were changed in PD striata compared to age-matched controls, Hsc70 levels were substantially reduced in cholate-insoluble fractions. This suggests that CMA may be impaired in PD.

This is one of the first studies showing impairments in autophhagic pathways in PD. Previously, a study found diminished Hsc 70 levels in *Substantia nigra* in PD post mortem tissues [46] while another study found decreases in Lamp-2 gene expression in the peripheral leukocytes of patients with sporadic Parkinson's disease [47]. As further research is conducted, the precise impairment and role that autophagy plays in PD will become evident, but it is clear.

There are only a small number of studies that have examined the effects of environmental toxins on tauopathy, even though tauopathy is a key feature common to most neurodegenerative diseases. Previously, rotenone was shown to cause widespread tauopathy in rat brain [48] and in cultured neurons of hippocampus, substantia nigra and locus coeruleus [49]. In both studies, increases in α-Syn levels were also found. Such studies, including our current one, clearly document the ability of agrichemicals to cause tauopathy that leads to neurodegeneration, resembling that seen in diseases of unknown etiology such as PD and AD. Paraquat remains one of the most widely used herbicides in the world and in an ever aging population its continued use may prove to be especially harmful, leading to a higher incidence of such diseases.

## Materials and Methods

### Materials

The antibodies used in this study are: anti-Tau MAB361 from Millipore [Temecula, CA]; anti-Tau Neurofibrillary Tangles Marker (Tau-5) AHB0042, anti-tau (pS262), and anti-Tau (pS396; immunohistochemistry only) from Biosource Invitrogen [Carlsbad, CA]; anti-α-Syn CAT# 610787, anti-GSK-3β CAT# 612313 and anti-pGSK-3β [purified mouse anti-GSK-3B (pY216) CAT# 612313], from BD Transduction Labs [San Jose, CA]; anti-β-actin SC-1616 from Santa Cruz Biotechnology, Inc. [Santa Cruz, CA]; the CP-13 and PHF-1 antibodies [recognizing Tau-Ser202 and Tau-Ser396/404, respectively] were gifts from Dr. Peter Davies [New York]; anti-Proteasome 19S Subunit S6' CAT# AP-111 and anti-Proteasome 20S Subunit α5 CAT# AP-120, from BostonBiochem [Cambridge, MA]; anti-Atg12 (D88H11) CAT# 4180, anti-Atg7 CAT# 2631, anti-LC3B (D11) XP CAT# 3868, anti-mTOR (7C10) CAT# 2983, anti-Hsp70 CAT# 4872, anti-Hsp90β CAT# 5087, from Cell Signaling Technology, Inc. [Danvers, MA]; anti-Beclin 1 CAT# NBP1-45382 from Novus Biologicals, LLC [Littleton, CO]; anti-beta Synuclein CAT# ab25650 and anti-Hsc70 [N27F34] CAT# ab90347 from Abcam PLC [Cambridge, MA]; and anti-α-Tubulin CAT# T6074 and anti-Acetylated-Tubulin CAT# T7451 from Sigma-Aldrich, Co. [St. Louis, MO].

### Human striata

Postmortem striatal tissue from PD and age-matched non-diseased controls were obtained and processed as described previously [25].

### Animals

Mice used in these studies were 2–3 month old males with a mixed C57BL/6×129S background. All studies with animals were conducted under strict guidelines of the National Institutes of Health and were approved by Georgetown University Animal Care and Use Committee.

### Treatment of mice with maneb and paraquat

Mice were injected essentially as described previously [10], [11]. Male mice on a mixed C57BL/6×129S background received intraperitoneal injections of 10 mg/kg paraquat (PQ) and/or 30 mg/kg maneb (MB) in sterile filtered 0.9% saline. Control mice were injected with saline alone (vehicle treated). Since PQ and MB were administered as separate injections, two separate injections of saline were administered to control mice, and a single injection of saline was administered to all single-toxin mice used as controls, so that all animals received the same number of injections and the same total volume of injected saline (adjusted for weight). Toxin injections were administered first, followed by saline injections. Treatment was administered twice weekly for six weeks (12 total injections) following which all animals were sacrificed. Striata were dissected for Western blot analysis.

### Determination of 26S proteasome activity

Striatal tissues were re-suspended in 10 vol of ice-cold extraction buffer (10 mM Tris–HCl, pH 7.4, 1 mM EDTA, 4 mM dithiothreitol, 20% glycerol) and disrupted by 50 strokes in a dounce homogenizer on ice. Lysates were cleared by centrifugation (20 minutes,16,000×g, 4°C). Soluble protein concentration was determined by Bio-Rad Protein Assay (Bio-Rad, Hercules, CA), and protein concentrations were equalized to 0.75 mg/ml by dilution in extraction buffer. For determination of 26S proteasome activity, 10 µl of lysate was combined with 85 µl of reaction buffer (20 mM Tris–HCl pH 7.4, 1 mM ATP, 20% glycerol) plus 5 µl of 0.1 mg/ml N-Succinyl-Leu-Leu-Val-Tyr-7-amino-4-methylcoumarin (suc-LLVY-AMC; Biomol, Plymouth Meeting, PA),and incubated at 37°C for 60 minutes. Reactions including 50 µM clasto-lactacystin β-lactone (lactacystin) 26S proteasome inhibitor were conducted in parallel. After incubation, reactions were transferred to opaque 96-well plates, and AMC fluorescence was measured on a Victor3V, 1420 Multi-label Counter (PerkinElmer, Waltham, MA) for 1 second, using an excitation filter of 355 nm and an emission filter of 460 nm. Lactacystin-resistant activity was subtracted from total activity to determine 26S activity.

### Preparation of lysates for Western blots

Tissues were homogenized in buffer containing 10 mM Tris-HCl, pH 7.4,1 M NaCl, 250 mM sucrose, 5 mM KCl, 2 mM CaCl_2_, 1 mM MgCl_2_, 1 mM dithiothreitol, 1 mM EDTA, 1 mM EGTA, 10 µg/ml each of phosphatase inhibitor cocktail (Halt Phosphatase inhibitor Cocktail; Thermo Scientific, Rockford, IL) and protease inhibitor cocktail tablets (Complete Mini, EDTA-free; Roche Diagnostics, Mannheim, Germany). Sodium cholate (20% in water, wt/vol) was added to a final concentration of 1% (vol/vol). The mixture was left on ice for 30 min with brief vortexing every 5 min, followed by centrifugation for 20 min at 16,000× *g* at 4°C. The clear supernatant representing cholate-soluble fractions, and pellets representing the cholate-insoluble fractions, were collected. Pellet fractions were briefly sonicated with a Branson Sonifier 250 and protein concentrations were measured using the Lowry assay. Samples were diluted to equal protein concentrations with homogenizing buffer, then further diluted 1∶4 with dilution buffer (50 mM Tris-HCl, pH 7.4, 5 mM KCl, 2 mM CaCl_2_, 1 mM MgCl_2_, 1 mM EDTA, 1 mM EGTA), so that final sodium cholate concentration was reduced to 0.2%. Samples were then mixed with Laemmli buffer [1∶1 vol/vol] and analyzed by Western blots.

### Isolation of cytoskeleton-free and cytoskeleton-associated fractions

Tissues were extracted and separated into cytoskeleton-free and cytoskeleton-associated fractions as described previously [22]. Briefly, tissues were homogenized in buffer [pre-warmed to room temperature] containing 80 mM PIPES (pH 6.8), 1 mM MgCl_2_, 2 mM EGTA, 0.1 mM EDTA, 0.1% Triton X-100 and 30% glycerol. Lysates were incubated at 37°C for 10 minutes prior to centrifugation at room temperature at 14,000× g for 20 min. The supernatant contained cytoskeleton-free fractions. The pellet, containing cytoskeleton-associated fraction, was re-suspended in 2% SDS, 5 mM EDTA, 5 mM EGTA, 10% glycerol, 0.25 M Tris-HCl (pH 6.8), incubated at room temperature on an inverter for 60 min, and sonicated 3×30 secs at room temperature with a Branson Sonifier 250.

### Western Blot Analysis

Western blot analysis was performed as described previously [26], [27]. Briefly, samples were analyzed on 10–20% Tris-HCl Criterion gels (Bio-Rad), after blocking with 20 mM Tris-buffered saline, pH 7.6 containing 0.1% Tween 20 (TBST) and 5% (wt/vol) blotting grade blocker non-fat dry milk (Bio-Rad) for 1 hour at room temperature. Western blots were probed with an array of primary antibodies diluted into TBST with 5% milk at experimentally determined concentrations ranging from 1∶500 to 1∶1000. After incubation for 2 hours at room temperature with HRP-conjugated secondary antibodies (1∶3000; Santa Cruz), proteins were revealed by enhanced chemiluminescence (Perkin Elmer). Images were scanned by Scanner EPSON Perfection V700 Photo and then quantified using ImageJ. Proteins were normalized to β-actin (1∶1000), used as loading control.

### Immunohistochemistry

IHC analysis of mouse brain coronal sections was performed as previously described [26], [27], with slight modifications. Briefly, brains from 2–3 month old saline treated and age-matched paraquat treated (10 mg/kg) mice were embedded in OTC and flash frozen in isopentane. 10 µm sections fixed to super-frost slides were washed, permeabilized, and stained in the following manner. Each slide was washed 3 times in 1 mg/mL NaBH_4_, 1× PBS pH 7.4, for 5 min at room temperature followed by washing 6×, for 10 min in 1× PBS pH 7.4, 1% Triton X-100, followed by blocking in the antibody specific blocking buffer for 1 h at room temperature in 1× PBS pH 7.4, 1% Triton X-100, 10% FCS. Incubation with primary antibody occurred at 4°C, overnight in the dark, in the appropriate blocking buffer using the following concentrations for sequential staining: mouse-anti-α-Syn, 1∶750; rabbit-anti-pSer396 Tau (pTau), 1∶500; rabbit-anti-mTOR, 1∶500. Following primary staining, each slice was washed 3× in 1× PBS pH 7.4, 1% Triton X-100 at room temperature, incubated for 30 min in blocking buffer with the appropriate Alexafluor 488 or 594 conjugated secondary antibody, and washed 5× in 1× PBS pH 7.4, 1% Triton X-100. Slides were mounted using Fluoromount-DAPI. Fluorescence images were captured using a laser scanning confocal microscope (Olympus FV300). Paired images between tissue from saline and paraquat treated animals for all figures were collected at the same laser power, gain, and offset settings. Post-collection processing with ImageJ, as well as contrast and color adjustment was applied uniformly to all paired images.

### Statistical Analysis

Results were expressed as mean ± SEM and statistically analyzed by the Student's t-test between two groups. Statistical significance was accepted at the [*p*<0.05] level.
