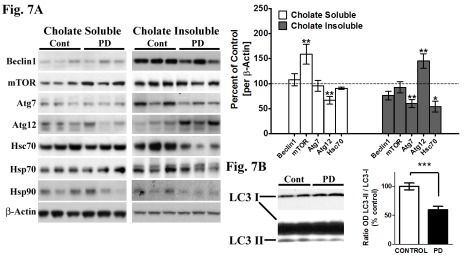# Correction: Paraquat, but Not Maneb, Induces Synucleinopathy and Tauopathy in Striata of Mice through Inhibition of Proteasomal and Autophagic Pathways

**DOI:** 10.1371/annotation/6c09a04c-e565-4a34-b24e-90f084463e15

**Published:** 2012-05-02

**Authors:** Jonathan Wills, Joel Credle, Adam W. Oaks, Valeriy Duka, Jae-Hoon Lee, Jessica Jones, Anita Sidhu

There were errors in Figures 1-7. The correct Figures can be viewed here:

Figure 1: 

**Figure:**
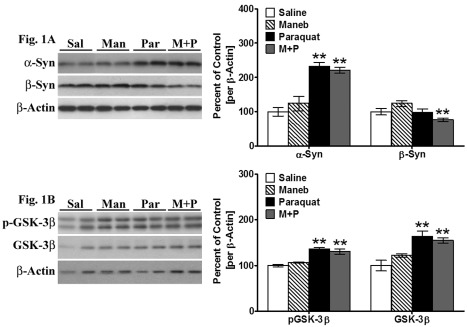


Figure 2: 

**Figure:**
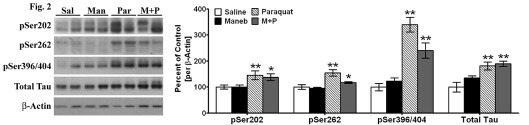


Figure 3: 

**Figure:**
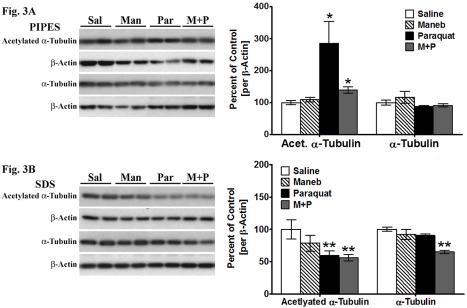


Figure 4: 

**Figure:**
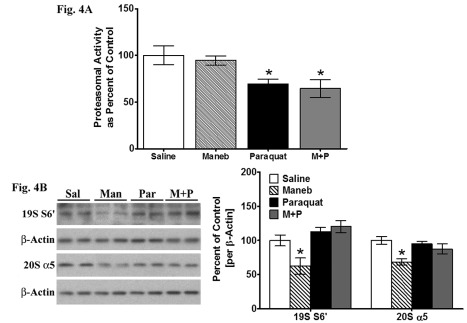


Figure 5: 

**Figure:**
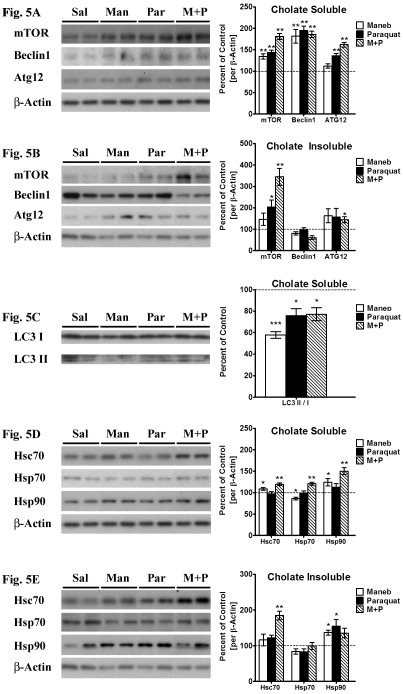


Figure 6: 

**Figure:**
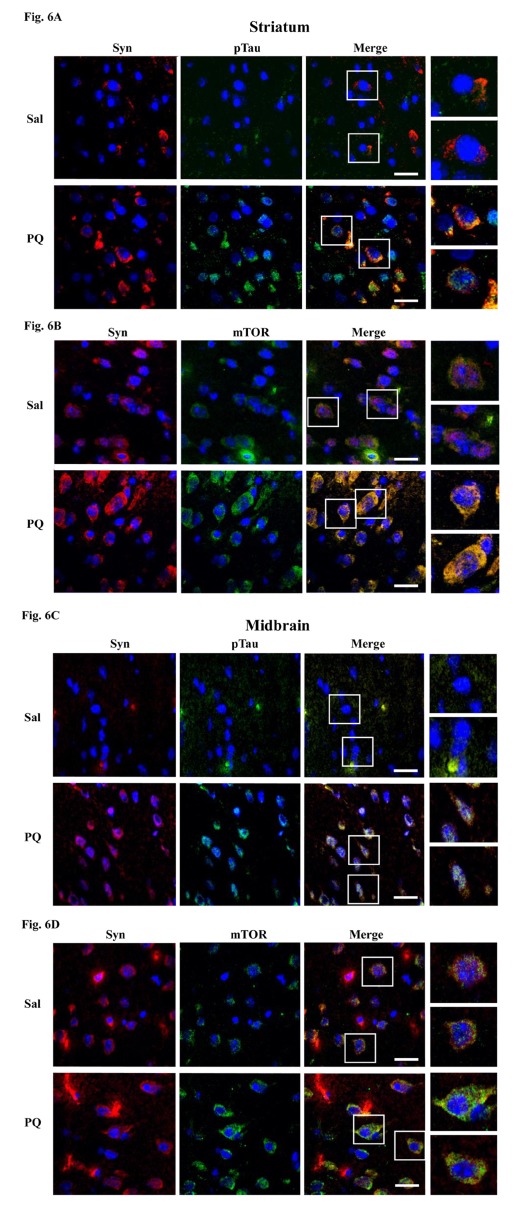


Figure 7: 

**Figure:**